# The genus *Alterosa* Blahnik, 2005 (Trichoptera, Philopotamidae, Philopotaminae) in northeastern Brazil, including the description of three new species and an identification key for the genus

**DOI:** 10.3897/zookeys.317.5437

**Published:** 2013-07-15

**Authors:** Leandro Lourenço Dumas, Adolfo Ricardo Calor, Jorge Luiz Nessimian

**Affiliations:** 1 Departamento de Zoologia, Instituto de Biologia, Universidade Federal do Rio de Janeiro, Caixa Postal 68044, Cidade Universitária, 21941-971, Rio de Janeiro, RJ, Brazil; 2 Universidade Federal da Bahia, Instituto de Biologia, Departamento de Zoologia, PPG Diversidade Animal, Laboratório de Entomologia Aquática - LEAq. Rua Barão de Jeremoabo, 147, campus Ondina, Ondina, CEP 40170-115, Salvador, Bahia, Brazil

**Keywords:** Atlantic Forest, caddisflies, distributional notes, Neotropical Region, taxonomy

## Abstract

*Alterosa* Blahnik, 2005 contains 35 described species distributed in southern and southeastern Brazil. Three new species of *Alterosa* from northeastern Brazil are described and illustrated, *Alterosa amadoi* **sp. n.**, *Alterosa castroalvesi* **sp. n.** and *Alterosa caymmii* **sp. n.**, the first records of the genus from northeastern Brazil. An identification key for all known species of the genus is also presented.

## Introduction

Philopotamidae are distributed throughout the world with 1,194 described species in 19 extant genera (Holzenthal et al. 2011). The family contains 3 subfamilies: Rossodinae, with the monotypic genus *Rossodes* Özdikmen & Darilmaz, 2008 limited to Madagascar, originally described as *Paulianodes* by Ross (1956); Chimarrinae, with nearly 600 species mainly in the cosmopolitan genus *Chimarra* Stephens, 1829; and the also cosmopolitan subfamily Philopotaminae, with more than 400 species (Blahnik 2004, 2005). The subfamily Philopotaminae contains 16 genera, most of relatively restricted distribution (Holzenthal et al. 2007). Only 3 genera of Philopotaminae are recorded from the Neotropics: *Sortosa* Navás, 1918, *Wormaldia* McLachlan, 1865, and *Alterosa* Blahnik, 2005 (Blahnik 2005).

The genus *Alterosa* was originally described by Blahnik (2005) to include 2 previously described species, *Dolophilodes (Sortosa) sanctipauli* Flint, 1971 and *Dolophilodes (Sortosa) marinonii* Almeida & Duarte, 2003, and 20 new species, totaling 22 species. More recently, 13 new species were described by Jardim and Dumas (2012) and Dumas and Nessimian (2013), raising the total to 35 species distributed exclusively in south and southeastern Brazil. These species are usually found in pristine headwaters and rapids of streams or small rivers in the Atlantic Forest; they are rarely encountered near large rivers. Despite the recent increase in the number of species known from the country, the Brazilian diversity of *Alterosa* seems to be greatly underestimated, with many more unknown species remaining to be collected. Additionally, there is a lack of knowledge of most aspects of the biology, ecology and distribution of the species, including their immature stages, which are unknown.

In this paper we provide descriptions, diagnoses and illustrations of 3 new species of *Alterosa* from Bahia state, the first records from northeastern Brazil. In addition, we provide an identification key for all known species of the genus.

## Material and methods

The specimens were collected with UV light pan traps (Calor and Mariano 2012) and preserved in 70% ethanol. In order to observe the genital structures, the abdomen of each specimen was removed and cleared in a heated lactic acid 85% solution, followed by a rinse in distilled water, transferred to a solution of 95% EtOH, and mounted temporarily in glycerin or glycerin jelly on a slide for viewing and drawing (Blahnik et al. 2007). After that, removed abdomens were transferred back to alcohol and stored permanently in micro vials with 80% EtOH. A Zeiss stereomicroscope and a Zeiss optical microscope, each equipped with a camera lucida, were used to observe specimens. Genitaia were drawn in pencil and drawings were inked with a technical pen and light table. Females were not illustrated because the minor differences among them were not diagnostic. The terminology used in the descriptions follows that of Blahnik (2005).

The type specimens and additional material were deposited in Coleção Entomológica Professor José Alfredo Pinheiro Dutra, Departamento de Zoologia, Universidade Federal do Rio de Janeiro, Rio de Janeiro state, Brazil (DZRJ), Museu de Zoologia da Bahia, Universidade Federal da Bahia, Bahia state, Brazil (UFBA), and Museu de Zoologia, Universidade de São Paulo, São Paulo state, Brazil (MZSP).

## Taxonomy

### Alterosa amadoi sp. n.

urn:lsid:zoobank.org:act:F0D50D4A-AE17-4732-BE5D-88C47B018FCF

http://species-id.net/wiki/Alterosa_amadoi

Figs 1A–D

#### Remarks.

*Alterosa amadoi* sp. n. cannot be easily placed in any of the species groups defined by Blahnik (2005). *Alterosa amadoi* sp. n. and *Alterosa caymmii* sp. n. share character similarities with *Alterosa ruschii* Dumas & Nessimian, 2013 and *Alterosa spiesae* Dumas & Nessimian, 2013, regarding the general structure of the branched intermediate appendages. However, unlike *Alterosa ruschii* and *Alterosa spiesae*, the 2 new species cannot be placed in or even near the *Alterosa sanctipauli* group because of the lack of a basal rounded protuberance on tergum X, with cuticle scabrously developed, and the absence of a crest-like process at the apex of tergum X. *Alterosa amadoi* sp. n. can be diagnosed by the overall shape of the intermediate appendages, especially the rod-like mesal branch with spines at its apex, and the inferior appendages with the 1st article short and nearly as wide as long. An additional diagnostically unique character for this new species is the dorsal spiny crest-like projection at midlength of tergum X.

#### Description.

Adult. Color (in alcohol) brown; legs, palps, and antennae pale brown, forewing pattern not discernible. Male forewings 5.6–6.4 mm (n=3).

**Figure 1.**
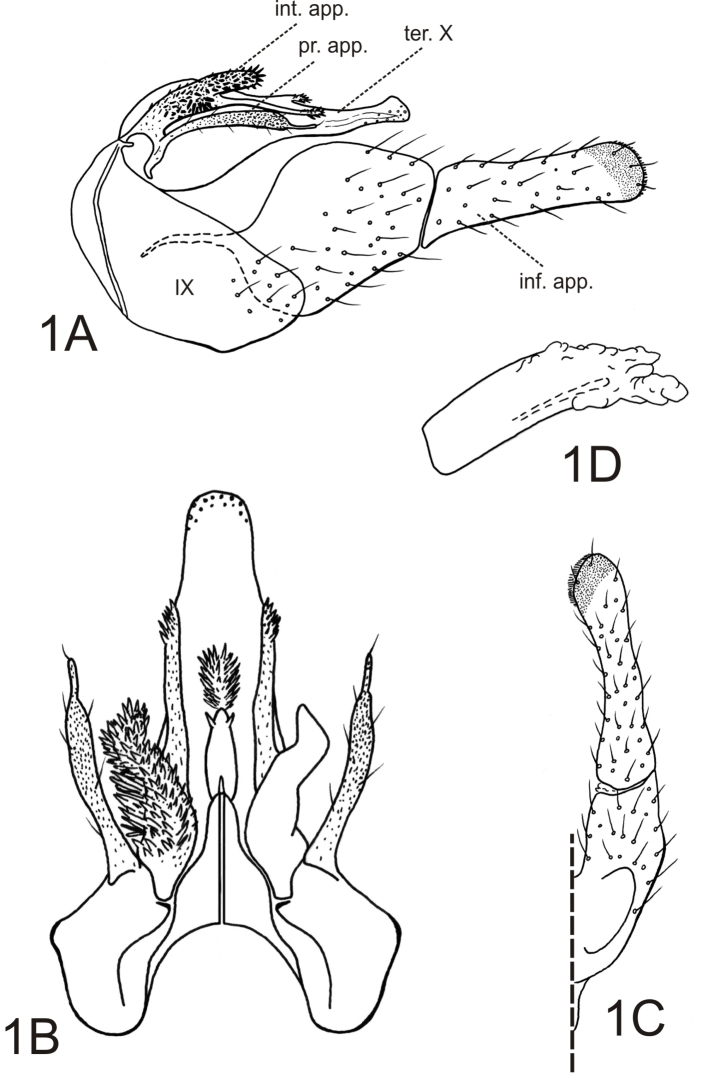
*Alterosa amadoi*, new species, male genitalia. **A** left lateral view **B** dorsal view (setae of left lateral branch of intermediate appendages omitted) **C** left inferior appendage, dorsal view **D** phallic apparatus, left lateral view. Abbreviations: inf. app. – inferior appendage; int. app. – intermediate appendage; pr. app. – preanal appendage; ter. X – tergum X; IX - tergum IX.

*Male genitalia*. Tergum VIII with posteromesal margin moderately emarginate; emargination U-shaped and extending no more than halfway to anterior margin. Sternum IX with anterolateral margin weakly rounded, subtruncate; posteroventral margin greatly produced, extending nearly linearly from dorsum (Fig. 1A). Tergum IX greatly reduced, membranous or fused to base of tergum X (Fig. 1B). Tergum X tapered from base in lateral view; dorsally with spiny crest-like projection at midlength; apex sensillate, subtruncate, slightly enlarged in lateral view (Figs 1A, 1B). Intermediate appendages heavily sclerotized, branched subbasally; mesal branch elongate, surpassing preanal appendages, rod-like, blunt and with small spines apically; lateral branch lobe-like, rounded, covered by spine-like setae, subacute apically in dorsal view, apex greatly enlarged and rounded as viewed laterally (Figs 1A, 1B). Preanal appendages elongate, club-like, covered with stiff, small setae; apex rounded with elongate subacuminate projection bearing small apical setae as viewed laterally and dorsally (Figs 1A, 1B). Inferior appendages robust, setose; 1st article, in lateral view, short, nearly as wide as long, bulging mesally; 2nd article longer than 1st article, relatively wide, base as wide as apex of 1st article, slightly enlarged apically and with small pad of short, stiff apicomesal setae (Figs 1A, 1C). Phallobase tubular, relatively short, slightly curved; endotheca without spines; phallotremal sclerites indistinct (Fig. 1D).

#### Materials.

**Holotype male: BRAZIL: Bahia:** Camacan, RPPN Serra Bonita, riacho 1, trilha nova, 15°23'35"S, 39°33'50"W, ca 770 m, bandeja, 1.iv.2011, F. Quinteiro, D. França & H. Barreto leg. (MZSP).

**Paratypes: BRAZIL: Bahia:** Camacan, RPPN Serra Bonita, riacho 1, trilha nova, 15°23'35"S, 39°33'50"W, ca 770 m, bandeja, 30.iii.2011, F. Quinteiro, D. França & H. Barreto leg., 1 male (DZRJ 3791); Camacan, RPPN Serra Bonita, 2ª cachoeira, over night, 03.xi.2009, A. R. Calor leg., 1 male (UFBA).

#### Distribution.

Brazil (Bahia).

#### Etymology.

The species is dedicated to the deceased Brazilian writer Jorge Amado, who was born in Bahia state in 1912 and died in 2001. Amado is one of the most acclaimed contemporary Brazilian novelists, whose 32 novels have sold millions of copies. Among his best-known works are “Capitães de Areia”, “Gabriela, Cravo e Canela”, and “Tieta do Agreste”. In 2012, Brazil celebrated the centenary of his birth.

### Alterosa castroalvesi sp. n.

urn:lsid:zoobank.org:act:04FB0169-E8AF-4E54-9F01-3DF278EA1B19

http://species-id.net/wiki/Alterosa_castroalvesi

Figs 2A–D

#### Remarks.

*Alterosa castroalvesi* sp. n. is closely related to *Alterosa truncata* Blahnik, 2005, resembling that species in the broadened and truncate apices of the inferior appendages, the elongate, narrow, and arched intermediate appendages, each terminating in a very prominent apical lance-like seta, and the general structure of preanal appendages. Additionally, the general structure of tergum X with paired longitudinal, serrate ridges, is also similar between these species. It differs in that the intermediate appendages in *Alterosa castroalvesi* sp. n. have 2 prominent lance-like setae, the usual apical 1 and an additional 1 at the apical third. *Alterosa castroalvesi* sp. n. also differs from *Alterosa truncata* in the length of 2nd article of preanal appendages, that is relatively shorter in the new species. The differences are relatively minor and it is possible that the 2 species may eventually prove to be not specifically distinct. As *Alterosa truncata*, the new species probably belongs to *Alterosa sanctipauli* Group, as defined by Blahnik (2005).

#### Description.

Adult. Color (in alcohol) brown; legs, palps, and antennae pale brown, forewing pattern not discernible. Male forewings 6.0–6.8 mm (n=4).

**Figure 2.**
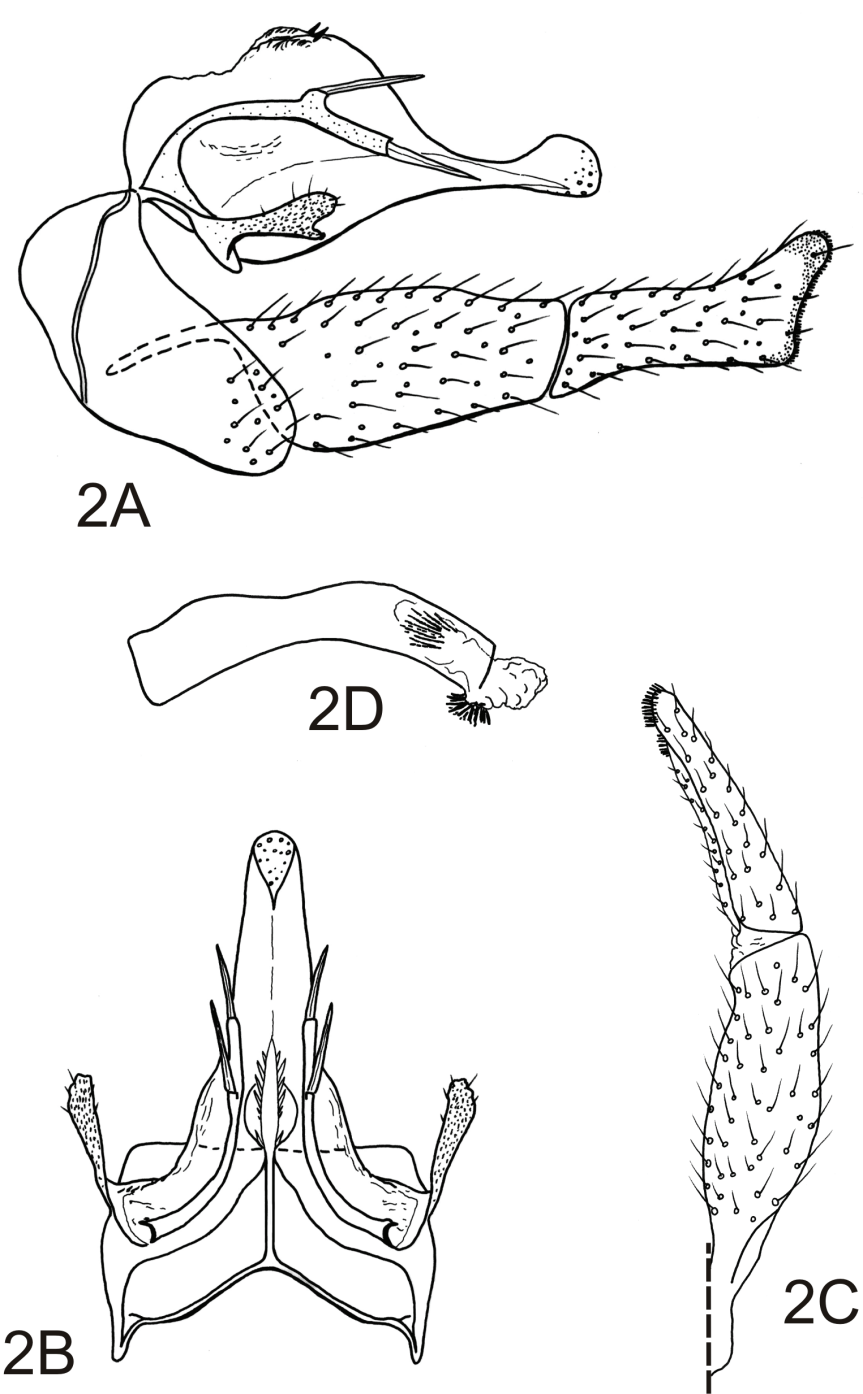
*Alterosa castroalvesi*, new species, male genitalia. **A** left lateral view **B** dorsal view **C** left inferior appendage, dorsal view **D** phallic apparatus, left lateral view.

*Male genitalia*. Tergum VIII with posteromesal margin deeply emarginate, emargination V-shaped and extending more than half way to anterior margin. Sternum IX with anterolateral margins broadly rounded; posteroventral margin greatly produced, forming broadly rounded expansion (Fig. 2A). Tergum IX greatly reduced, membranous or fused to base of tergum X (Fig. 2B). Tergum X narrow, wider at base, lateral margins subparallel in dorsal view; dorsally with mesally divided crest-like projection near base, with spine-like projections along each side of divided margin; apex sensillate, rounded as viewed dorsally, rounded and slightly enlarged in lateral view (Figs 2A, 2B). Intermediate appendages heavily sclerotized, elongate, extending past preanal appendages, rod-like, curved at base, narrowly paralleling lateral margins of tergum X; intermediate appendages with small projection at apical third bearing 1 apical lance-like seta; apex acute, terminating in apical lance-like seta (Figs 2A, 2B). Preanal appendages narrow, constricted basally, moderately elongate, not greatly modified, covered with scant setae and numerous minute spine-like setae; apex rounded, without apical setae, with 1 short preapical projection from the ventral surface, fringed apically with minute spines (Figs 2A, 2B). Inferior appendages elongate, linear, flattened on mesal surface, setose; each with 1st article, in lateral view, approximately 2 times as long as wide, tapering apically; 2nd article slightly shorter than 1st article, moderately wide, nearly as wide at base as apex of 1st article, apex enlarged and subtruncate, with fringing pad of short, stiff apicomesal setae (Figs 2A, 2C). Phallobase tubular, narrow, moderately elongate, with slightly curvature; endotheca with 2 tracts of fine spines, 1 of needle-like spines and 1 ventral of shorter spines (Fig. 2D).

#### Materials.

**Holotype male: BRAZIL: Bahia:** Camacan, Serra Bonita, Córrego das Torres, 15°23'01"S, 39°34'19"W, ca 860 m, 02.viii.2008, A. R. Calor, L. Lecci, L. C. Pinho & A. Moretto leg. (MZSP).

**Paratypes: BRAZIL: Bahia:** same data as holotype, 2 males (DZRJ 3797), 1 male (UFBA).

#### Distribution.

Brazil (Bahia).

#### Etymology.

This species is named in memory of Antônio Frederico de Castro Alves, known as “the poet of the slaves” because of his sympathy for the Brazilian abolitionist cause. Castro Alves was born in Bahia state in 1847 and died at 1871. He is the patron of the 7th chair of the Brazilian Academy of Letters. Some of his abolitionist poems, like “Espumas Flutuantes”, “A Cachoeira de Paulo Afonso”, and “O Navio Negreiro”, were collected in a posthumous book called “Os Escravos”, published in 1883.

### Alterosa caymmii sp. n.

urn:lsid:zoobank.org:act:51728D88-1218-4C1A-ADDF-FED6872BBE77

http://species-id.net/wiki/Alterosa_caymmii

Figs 3A–F

#### Remarks.

*Alterosa caymmii* sp. n. is very similar to *Alterosa amadoi* sp. n., as discussed under that species. It differs in that the lateral branch of each intermediate appendage is apically projecting in *Alterosa caymmii* sp. n., while in *Alterosa amadoi* sp. n. it is broadly rounded. *Alterosa caymmii* sp. n. also differs from *Alterosa amadoi* sp. n. in the shape of inferior appendages. Additionally, the phallus of *Alterosa caymmii* sp. n. is more developed, with endotheca bearing several tracts of numerous large spines.

#### Description.

Adult. Color (in alcohol) brown; legs, palps, and antennae pale brown, forewing pattern not discernible. Male forewings 5.2–6.4 mm (n=10).

**Figure 3.**
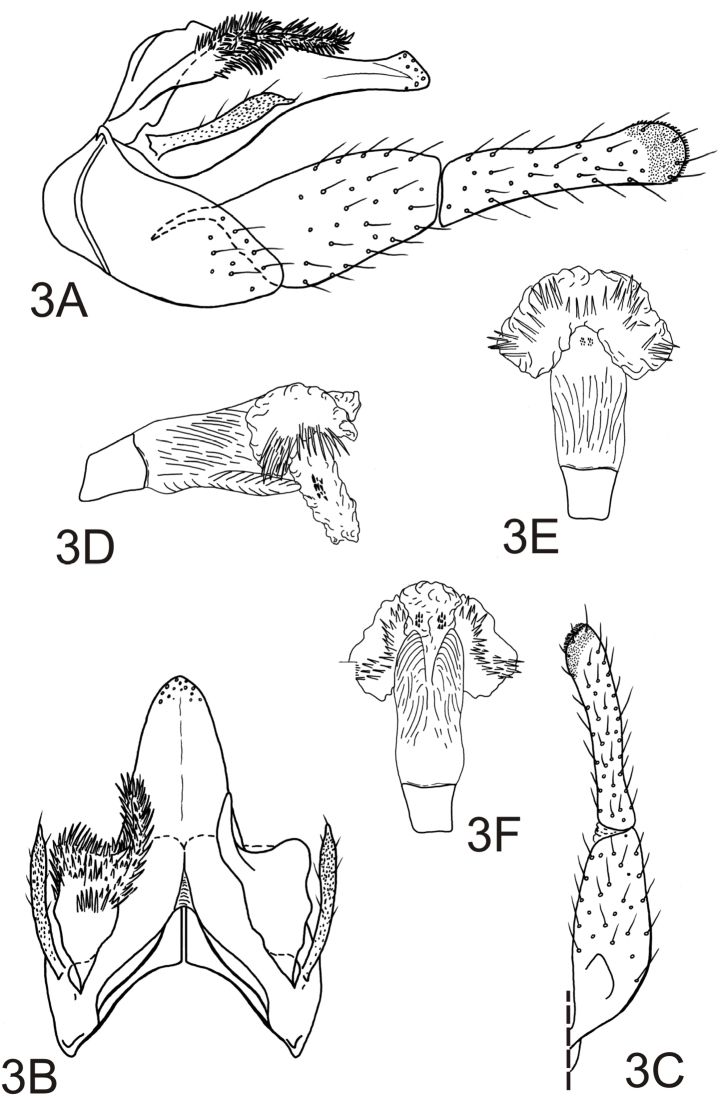
*Alterosa caymmii*, new species, male genitalia. **A** left lateral view **B** dorsal view **C** left inferior appendage, dorsal view (setae of left lateral branch of intermediate appendages omitted) **D** phallic apparatus, left lateral view **E** phallic apparatus, dorsal view; 3F, phallic apparatus, ventral view.

*Male genitalia*. Tergum VIII with posteromesal margin not or scarcely emarginate. Sternum IX with anterolateral margins weakly rounded, subtruncate; posteroventral margin greatly produced, forming broadly rounded expansion (Fig. 3A). Tergum IX greatly reduced, membranous or fused to base of tergum X (Fig. 3B). Tergum X tapered from base in lateral view; apex sensillate, rounded as viewed dorsally, subtruncate and slightly enlarged in lateral view (Figs 3A, 3B). Intermediate appendages heavily sclerotized, branched subbasally; mesal branch moderately elongate, surpassing preanal appendages, slightly curved outward at midlength in dorsal view, rod-like, rounded apically, covered with spine-like setae; lateral branch short, flange-like, covered with spine-like setae in apical half, apex enlarged and rounded in lateral and dorsal views (Figs 3A, 3B). Preanal appendages elongate, club-like, covered with stiff, small setae; apex rounded with small subacuminate projection bearing small apical setae as viewed laterally and dorsally (Figs 3A, 3B). Inferior appendages elongate, linear, flattened on mesal surface, setose; each with 1st article, in lateral view, approximately 2 times as long as wide, tapering apically; 2nd article subequal in length to 1st article, relatively narrow, especially at midlength, apex rounded, with prominent pad of short, stiff apicomesal setae (Figs 3A, 3C). Phallobase tubular, very short, scarcely curved; endotheca elongate, with several tracts; 1 paired tract near base without spines, 1 apicolateral tract with several large spines, and 1 single tract at apex with 2 clusters of small spines; phallotremal sclerites indistinct (Figs 3D, 3E, 3F).

#### Materials.

**Holotype male: BRAZIL: Bahia:** Varzedo, Serra da Jibóia, Reserva Jequitibá, 12°52'21.5"S, 39°28'56.5"W, 07.xi.2010, ca 400 m, A. M. Silva-Neto & M. Araújo leg. (MZSP).

**Paratypes: BRAZIL: Bahia:** same data as holotype, 7 males, 3 females (UFBA); Varzedo, Reserva Gambá, 12°57'12"S, 39°28'32"W, ca 510 m, 08.xi.2010, bandeja, A. R. Calor, F. Quinteiro, D. França, R. Mariano & A. Costa leg., 17 males, 1 female (UFBA); Santa Teresinha, Serra da Jibóia, Riacho das Torres, 12°50'59"S, 39°28'47"W, ca 490 m, 06.x.2010, Luz, A. R. Calor, F. Quinteiro, D. França, R. Mariano & A. Costa leg., 37 males, 16 females (DZRJ 3792); Santa Teresinha, Distrito de Pedra Branca, Riacho das Torres, 12°51'00"S, 39°28'48"W, ca 490 m, 28.ix.2009, Luz, A. R. Calor & A; Cruz leg., 1 male (UFBA); Santa Teresinha, Distrito de Pedra Branca, Riacho das Torres, 12°51'00"S, 39°28'48"W, ca 490 m, 10.vi.2010, Luz, A. R. Calor, D. França & F. B. Quinteiro leg., 1 male (UFBA); Santa Teresinha, Distrito de Pedra Branca, Riacho das Torres, 12°51'01"S, 39°28'48"W, ca 500 m, 07.viii.2009, A. R. Calor & L. Lecci leg., 1 male (UFBA); Varzedo, Serra da Jibóia, Fazenda Baixa da Areia Cai Camarão, 12°57'45"S, 39°27'12"W, ca 260 m, 27.iii.2012, Pan light trap, F. B. Quinteiro, T. Duarte & I. Garcia leg., 2 males (UFBA); Camacan, Serra Bonita, córrego 1, 15°23'28"S, 39°33'56"W, ca 820 m, 31.vii.2008, Luz UV e branca, A. R. Calor, L. Lecci, L. C. Pinho & R. Moretto leg., 2 males, 1 female (DZRJ 3793); Camacan, Serra Bonita, 1ª cachoeira, 15°23'28"S, 39°33'57"W, ca 830 m, 04.xi.2009, A. R. Calor et al. leg., 2 males (UFBA); Camacan, Fazenda Waldemar da farmácia, 15°25'17"S, 39°34'01"W, ca 310 m, 28.iii.2011, A. R. Calor, F. Quintero, D. França & H. Barreto leg., 1 male (DZRJ 3794); Camacan, RPPN Serra Bonita, riacho 1, 15°23'40"S, 39°33'44"W, ca 720 m, 31.iii.2011, F. Quinteiro, D. França & H. Barreto leg., 2 males (UFBA); Camacan, RPPN Serra Bonita, Malaise 1, 15°23'28"S, 39°33'56"W, ca 260 m, iv.2011, A. R. Calor leg., 5 males, 2 females (DZRJ 3795); Wenceslau Guimarães, Estação Ecológica Estadual Wenceslau Guimarães, Riacho Serra Grande, cachoeira em cima, 13°35'43"S, 39°43'12"W, ca 580 m, 10.x.2010, A. R. Calor et al. leg., 2 males, 2 females (UFBA); Wenceslau Guimarães, Estação Ecológica Estadual Wenceslau Guimarães, Rio Patioba, 13°34'50"S, 39°42'17"W, ca 540 m, 09.x.2010, A. R. Calor et al. leg., 1 male (DZRJ 3796); Amargosa, Serra do Timbó, Fazenda Timbó, Córrego Santa Rita, 13°06'22"S, 39°39'59"W, ca. 710 m, 17.vii.2009, A. R. Calor & L. Lecci leg.,1 male (UFBA).

#### Distribution.

Brazil (Bahia).

#### Etymology.

This species is named in memory of Dorival Caymmi, considered one of the most important Brazilian songwriters. Caymmi was born in Bahia state in 1914 and died at 2008. He became a national icon with his lyrics that evoked the charm of Bahia’s fishing villages, beaches and beautiful women, like “O Que é Que a Baiana Tem?”, “Marina”, “Rainha do Mar”, “Samba da Minha Terra”, among others.

### Key to species of *Alterosa* (males)

**Table:** 

1	Intermediate appendages curved, sickle-like (see figs 10A, 18A in Blahnik 2005)	2
–	Intermediate appendages apparently absent or not sickle-like	6
2	Tergum X with cuticle mesally rough, covered by numerous spines; endotheca of phallus without prominent spines (see figs 9A, 9B, 9D in Dumas and Nessimian 2013)	*Alterosa morato* Dumas & Nessimian
–	Tergum X without spiny cuticle; endotheca of phallus with small number of large spines (see figs 18A, 18B, 18D in Blahnik 2005)	3
3	Tergum IX short, forming a mesal projection over base of tergum X (see figs 2, 3 in Jardim and Dumas 2012)	4
–	Tergum IX greatly reduced, membranous or fused to base of tergum X	5
4	Intermediate appendages with 2 small spines; inferior appendages enlarged at apex (see figs 2, 3 in Jardim and Dumas 2012)	*Alterosa nessimiani* Jardim & Dumas
–	Intermediate appendages without spines; inferior appendages not enlarged at apex (see figs 18A, 18B in Blahnik 2005)	*Alterosa jordaensis* Blahnik
5	Tergum X dorsally with small preapical projection, forming small prominence; preanal appendages robust, surpassing the intermediate appendages (see figs 1A, 1B in Dumas and Nessimian 2013)	*Alterosa affinis* Dumas & Nessimian
–	Tergum X dorsally with variable small projection, never preapical (absent in some specimens); preanal appendages not surpassing the intermediate appendages (see figs 10A, 10B in Blahnik 2005)	*Alterosa falcata* Blahnik
6	Intermediate appendages absent	7
–	Intermediate appendages present	8
7	Tergum X with basoventral paired spine-like projection; tergum X with paired bristle-like setae; preanal appendages present (see figs 7A, 7B in Dumas and Nessimian 2013)	*Alterosa graciosa* Dumas & Nessimian
–	Tergum X with no basoventral projection nor bristle-like setae; preanal appendages absent (see figs 8A, 8B in Dumas and Nessimian 2013)	*Alterosa inappendiculata* Dumas & Nessimian
8	Intermediate appendages forming a short, rounded, knob-like, spinose processes (see figs 24B, 25B in Blahnik 2005)	9
–	Intermediate appendages elongate or moderately elongate, not forming a rounded, knob-like, spinose processes	11
9	Tergum IX forming mesal, hood-like projection over base of tergum X; tergum X with not basolateral expansions nor apical crest-like process (see figs 25A, 25B in Blahnik 2005)	*Alterosa tripuiensis* Blahnik
–	Tergum IX greatly reduced, membranous or fused to base of tergum X; tergum X basolaterally with broadly rounded wing-like expansions on either side and with longitudinally narrowed crest-like process at apex (see figs 10A, 10B in Dumas and Nessimian 2013)	10
10	Preanal appendages with subacuminate projection apicoventrally; inferior appendages rounded apically (see figs 10A, 10B in Dumas and Nessimian 2013)	*Alterosa paranaensis* Dumas & Nessimian
–	Preanal appendages without subacuminate projection apicoventrally; inferior appendages subtruncate apically (see figs 24A, 24B in Blahnik 2005)	*Alterosa schadrackorum* Blahnik
11	Preanal appendages apically with oblique concavity bearing a stout, spine-like seta (see figs 14A, 14B in Blahnik 2005)	12
–	Preanal appendages without oblique concavity at apex	13
12	Tergum X with lateral margins subparallel, not constricted as viewed dorsally; intermediate appendages rounded at apex, with apical small spines (see figs 14A, 14B in Blahnik 2005)	*Alterosa guapimirim* Blahnik
–	Tergum X constricted at apical third as viewed dorsally; intermediate appendages concave at apex, with 1 or 2 spine-like small setae (see figs 20A, 20B in Blahnik 2005)	*Alterosa orgaosensis* Blahnik
13	Tergum X with rounded basal protuberance, usually bearing scabrous cuticle; tergum X with longitudinally narrowed crest-like process at apex (see figs 5A, 7A, 11A in Blahnik 2005)	14
–	Tergum X with neither basal protuberance nor crest-like process at apex	24
14	Intermediate appendages branched basally or subbasally (see figs 4A, 12A in Dumas and Nessimian 2013)	15
–	Intermediate appendages single, not branched	20
15	Tergum X dorsally with paired longitudinal rows of short spines (see figs 7A, 7B in Blahnik 2005)	16
–	Tergum X without longitudinal rows of spines	17
16	Intermediate appendages with lateral branch very short, positioned laterally to mesal branch; intermediate appendages with lateral and mesal branches bearing apical brush of setae (see figs 7A, 7B in Blahnik 2005)	*Alterosa boraceiae* Blahnik
–	Intermediate appendages with lateral branch elongate, positioned below mesal branch; intermediate appendages with lateral and mesal branches covered with modified long, stout spine-like setae (see figs 4A, 4B in Dumas and Nessimian 2013)	*Alterosa caissara* Dumas & Nessimian
17	Tergum X apically covered with numerous elongate spine-like setae (see figs 8A, 8B in Blahnik 2005)	*Alterosa caparaonensis* Blahnik
–	Tergum X without spine-like setae at apex	18
18	Tergum X with apicodorsal crest-like Y-shaped expansion; preanal appendages short, not surpassing the lateral branch of intermediate appendages; intermediate appendages with lateral branch lobe-like, laterally compressed (see figs 11A, 11B in Dumas and Nessimian 2013)	*Alterosa ruschii* Dumas & Nessimian
–	Tergum X without crest-like expansion at apex; preanal appendages elongate, at least equal in length to lateral branch of intermediate appendages; intermediate appendages with lateral branch rod-like, not compressed	19
19	Preanal appendages abruptly narrowing at apex, forming subacuminate projection; mesal branch of intermediate appendages almost equal in length to lateral branch (see figs 12A, 12B in Dumas and Nessimian 2013)	*Alterosa spiesae* Dumas & Nesssimian
–	Preanal appendages not forming subacuminate projection apically; mesal branch of intermediate appendages much longer than lateral branch (see figs 5A, 5B in Blahnik 2005)	*Alterosa beckeri* Blahnik
20	Preanal appendages elongate, thick, stout, apically enlarged and with brush of setae (see figs 17A, 17B in Blahnik 2005)	*Alterosa itatiaiae* Blahnik
–	Preanal appendages short or moderately elongate, narrow, apex usually narrowed (see figs 11A, 23A in Blahnik 2005)	21
21	Intermediate appendages narrow, rod-like, elongate, apically with spine-like setae (see figs 6A, 11A in Blahnik 2005)	22
–	Intermediate appendages wide, short, apically broadened, covered by many coarse setae (see figs 16A, 23A in Blahnik 2005)	23
22	Tergum X dorsally with paired longitudinal rows of short spines, apex truncate in lateral view; intermediate appendages with preapical row of about 4 elongate spine-like setae (see figs 16A, 16B in Blahnik 2005)	*Alterosa intervales* Blahnik
–	Tergum X dorsally without longitudinal rows of short spines, apex rounded in lateral view; intermediate appendages with 2 or 3 small apical spine-like setae (see figs 23A, 23B in Blahnik 2005)	*Alterosa sanctipauli* (Flint)
23	Tergum X dorsally with paired longitudinal rows of short spines; intermediate appendages without long basal stalk, broadly expanded at apex (see figs 11A, 11B in Blahnik 2005)	*Alterosa fimbriata* Blahnik
–	Tergum X dorsally without longitudinal rows of short spines; intermediate appendages with long basal stalk, moderately expanded at apex (see figs 6A, 6B in Blahnik 2005)	*Alterosa bocainae* Blahnik
24	Inferior appendages truncate or subtruncate apically	25
–	Inferior appendages rounded apically	27
25	Tergum X dorsally with mesally divided spinose crest-like projection near base; preanal appendages club-like, moderately elongate, not surpassing tergum X; intermediate appendages rod-like, arched, with 1 or 2 lance-like setae (see Figs 2A, 2B)	26
–	Tergum X without crest-like projection basally; preanal appendages thick, rod-like, very elongate, about same length of tergum X; intermediate appendages pencil-like, glabrous (see figs 5A, 5B in Dumas and Nessimian 2013)	*Alterosa capixaba* Dumas & Nessimian
26	Intermediate appendages with 1 apical lance-like seta; inferior appendages with 2nd article subequal in length to 1st article (see figs 26A, 26B in Blahnik 2005)	*Alterosa truncata* Blahnik, 2005
–	Intermediate appendages with 2 lance-like setae, 1 at apical third and 1 at apex; inferior appendages with 2nd article slightly shorter than 1st article (Figs 2A, 2B)	*Alterosa castroalvesi* sp. n.
27	Preanal appendages greatly modified and enlarged, widened basally, armed with stout, modified setae (see figs 13A, 19A, 22A in Blahnik 2005)	28
–	Preanal appendages not enlarged basally, without stout setae	33
28	Intermediate appendages branched basally, with mesal branch spine-like (see figs 12B, 22B in Blahnik 2005)	29
–	Intermediate appendages single, not branched	31
29	Preanal appendages club-like, elongate, surpassing the intermediate appendages, with a basal enlargement, not forming a process (see figs 2A, 2B in Dumas and Nessimian 2013)	*Alterosa bandeira* Dumas & Nessimian
–	Preanal appendages bulbous, moderately elongate, not surpassing the intermediate appendages, with mesal process or dorsal process (see figs 12A, 22A in Blahnik 2005)	30
30	Preanal appendages with a mesal spinose pad-like process, without dorsal lobe (see figs 22A, 22B in Blahnik 2005)	*Alterosa sanctateresae* Blahnik
–	Preanal appendages without mesal process, forming a greatly enlarged, rounded dorsal lobe process, laterally compressed (see figs 12A, 12B in Blahnik 2005)	*Alterosa flinti* Blahnik
31	Preanal appendages club-like, without process basally (see figs 19A, 19B in Blahnik 2005)	*Alterosa marinonii* (Almeida & Duarte)
–	Preanal appendages subtriangular, with a flange-like process at base (see figs 9A, 13A in Blahnik 2005)	32
32	Tergum IX forming a mesal projection over base of tergum X (in some specimens with small, stalked basal process); preanal appendages with short, stout setae apically, extending along ventromesal margin; intermediate appendages glabrous (see figs 13A, 13B in Blahnik 2005)	*Alterosa fluminensis* Blahnik
–	Tergum IX greatly reduced, membranous or fused to base of tergum X; preanal appendages brush-like, with short, stout setae confined to posterolateral margin; intermediate appendage with scale-like spines along dorsal margin (see figs 9A, 9B in Blahnik 2005)	*Alterosa escova* Blahnik
33	Inferior appendages with 2nd article longer than 1st article	34
–	Inferior appendages with 2nd article subequal in length or shorter than 1st article	37
34	Intermediate appendages not branched, narrow, pencil-like, closely adpressed to lateral margin of tergum X, with a short, stout apical setae (see figs 15B, 21B in Blahnik 2005)	35
–	Intermediate appendages branched subbasaly, large, covered by stout, spine-like setae	36
35.	Tergum VIII with paired rod-like forked projections; tergum IX with lateral margins forming elongate dorsoventrally flattened plates (see figs 21A, 21B in Blahnik 2005)	*Alterosa paprockii* Blahnik
–	Tergum VIII without projections; tergum IX forming a shelf-like projection over base of tergum X (see figs 15A, 15B in Blahnik 2005)	*Alterosa holzenthali* Blahnik
36	Tergum X dorsally with spiny crest-like projection at midlength; intermediate appendages with lateral branch lobe-like; inferior appendages with 1st article short, nearly as wide as long; endotheca of phallus without prominent spines (Figs 1A, 1B, 1D)	*Alterosa amadoi* sp. n.
–	Tergum X without crest-like projection; intermediate appendages with lateral branch flange-like; inferior appendages with 1st article approximately 2 times as long as wide; endotheca of phallus with several paired tracts bearing prominent spines (Figs 3A, 3B, 3D, 3E, 3F)	*Alterosa caymmii* sp. n.
37	Tergum X robust, abruptly tapered subapically; intermediate appendages bent downward beneath tergum X, branched basally; phallobase without projections (see figs 6A, 6B, 6D in Dumas and Nessimian 2013)	*Alterosa catarinae* Dumas & Nessimian
–	Tergum X narrow, enlarged at base, with margins weakly protruding, subparallel; intermediate appendages rod-like, not bent, unbranched; phallobase with 2 long spear-like projections on posteroventral margin (see figs 3A, 3B, 3D, 3E in Dumas and Nessimian 2013)	*Alterosa bilanceolata* Dumas & Nessimian

## Supplementary Material

XML Treatment for Alterosa amadoi

XML Treatment for Alterosa castroalvesi

XML Treatment for Alterosa caymmii
